# Scarless and site-directed mutagenesis in *Salmonella enteritidis *chromosome

**DOI:** 10.1186/1472-6750-7-59

**Published:** 2007-09-17

**Authors:** Mandy M Cox, Sherryll L Layton, Tieshan Jiang, Kim Cole, Billy M Hargis, Luc R Berghman, Walter G Bottje, Young Min Kwon

**Affiliations:** 1 Department of Poultry Science, University of Arkansas, Fayetteville, AR, USA; 2 Department of Poultry Science and Veterinary Pathobiology, Texas A&M University, College Station, TX, USA

## Abstract

**Background:**

A variety of techniques have been described which introduce scarless, site-specific chromosomal mutations. These techniques can be applied to make point mutations or gene deletions as well as insert heterologous DNA into bacterial vectors for vaccine development. Most methods use a multi-step approach that requires cloning and/or designing repeat sequences to facilitate homologous recombination. We have modified previously published techniques to develop a simple, efficient PCR-based method for scarless insertion of DNA into *Salmonella enteritidis *chromosome.

**Results:**

The final product of this mutation strategy is the insertion of DNA encoding a foreign epitope into the *S. enteritidis *genome without the addition of any unwanted sequence. This experiment was performed by a two-step mutation process via PCR fragments, Red recombinase and counter-selection with the I-SceI enzyme site. First, the I-SceI site and kanamycin resistance gene were introduced into the genome of cells expressing Red recombinase enzymes. Next, this sequence was replaced by a chosen insertion sequence. DNA fragments used for recombination were linear PCR products which consisted of the foreign insertion sequence flanked by homologous sequences of the target gene. Described herein is the insertion of a section of the M2e epitope (LM2) of Influenza A virus, a domain of CD154 (CD154s) or a combination of both into the outer membrane protein LamB of *S. enteritidis*.

**Conclusion:**

We have successfully used this method to produce multiple mutants with no antibiotic gene on the genome or extra sequence except those nucleotides required for expression of epitope regions. This method is advantageous over other protocols in that it does not require cloning or creating extra duplicate regions to facilitate homologous recombination, contains a universal construct in which an epitope of choice can be placed to check for cell surface expression, and shows high efficiency when screening for positive mutants. Other opportunities of this mutational strategy include creating attenuated mutants and site-specific, chromosomal deletion mutations. Furthermore, this method should be applicable in other gram-negative bacterial species where Red recombinase enzymes can be functionally expressed.

## Background

Scarless, site-directed mutagenesis on a bacterial chromosome is often a preferred method for studying a particular region of DNA. This is due to the locational relevance and stability of the construct. The approach of constructing mutations on plasmids is still used consistently for many applications but is not always appropriate in the case of deletion mutations and vaccine therapy. In the case of live bacterial vaccines, inserting heterologous antigens on surface expressed proteins of bacteria has provided an efficient means to display immunogenic antigens. However, using plasmids for expression of these antigens comes with the risk of posing a metabolic burden on the bacterial cell, which causes decreased fitness or loss of the plasmid.

Still, several challenges exist for bacterial genomic mutagenesis and include the following: designing and creating delivery vectors that carry target genes with desired modifications, overcoming the host restriction system, avoiding the cause of a polar effect on downstream sequences, and eliminating unwanted scar sequences or antibiotic genes on the genome. All of these challenges have been confronted and either completely or partially overcome using a set of similar techniques. For example, a mutational strategy using Red recombinase was introduced in *Escherichia coli *as well as in *Salmonella typhimurium *which resulted in site-directed, chromosomal insertions or deletions but still had the problem of extraneous DNA left behind on the genome [1,2]. Additionally, another method was designed in which foreign epitopes were added to the C-terminal end of genes in *S. typhimurium*, yet, as before, an antibiotic gene or FRT scar sequence will remain on the genome [3]. More desirable would be a design scheme which would allow the epitope insertion at any location within a gene but without any additional scar sequence remaining. Therefore, improvements to this method were made, using the Sce-I endonuclease as a counter-selection tool, which could produce a scarless mutation and eliminate extraneous DNA or antibiotic resistance genes in the final mutational construct [4,5]. By a stimulated, double-strand break of the DNA, markerless mutations were constructed on BAC clones [5] as well as on the *E. coli *genome [6]. Yet, these protocols using Sce-I counter-selection require cloning and designing repeat sequences flanking the DNA of interest. The repeat sequences are the key elements of intramolecular recombination, and, in some cases, the variability of chromosomal recombination requires extra screening by PCR to determine which clones are positive mutants. Finally, a transposon-based method has been established which also uses Red recombinase and Sce-I counter-selection to construct site-directed mutations but without the need for cloning or designing repeat sequences [6]. This protocol for transposon mutagenesis requires amplifying open reading frames of genes from *E. coli *and performing *in vitro *transposition using these PCR products. Because PCR products ranged from 102–4617 bp in length, the transposon could be flanked by approximately 50–2300 bp of homology to the corresponding genomic site. The flanking sequences provided by PCR products reduce the need for designing sequences to facilitate recombination and also has the potential for providing greater lengths of homology. This might be more important for organisms, other than *E. coli*, to overcome unique restriction systems. For example, homologies ranging from 36–50 nucleotides are significant enough to allow recombination in *E. coli *by Red recombinase [2], but it has been reported that *Salmonella enteritica *serovar Enteritidis (*S. enteritidis*) may require 100 bp – 1 kb of sequence homology for recombination to be efficient [7].

Presently, we have applied techniques from the afore mentioned transposon-based method that allows scarless mutations [6] with an overlapping extension PCR strategy to chauffeur linear DNA fragments into a specific location on a bacterial genome [8]. PCR products were designed to carry insertion sequences flanked by 200–300 bp of homology to the target site on the chromosome. The resulting method uses the tailored-designed PCR products to conduct a two-step, site-directed mutation inserting a specific nucleotide sequence into the *S. enteritidis *genome without leaving a scar. In the present study we inserted a section of the M2e epitope (LM2) [9] from influenza A virus, a domain of CD154 (CD154s) [10], also known as CD40 ligand, or a combination of both into the outer membrane protein *lamB *gene of *S. enteritidis *in order to investigate the potential for a Salmonella-based vaccine against avian influenza virus.

## Results and discussion

### Overview of mutational strategy

**Figure 1:**
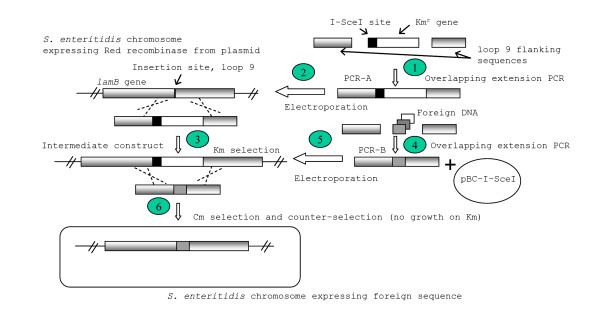
Overall design scheme for making site-directed, mutations by inserting foreign DNA into *S. enteritidis *genome; Step 1: Overlapping extension PCR is used to construct PCR-A product consisting of the I-SceI recognition sequence and Km^r ^gene flanked by homologous regions upstream and downstream to the insertion site in loop 9 of lamB in *Salmonella enteritidis*. Step 2: PCR-A is introduced into the chromosome by electroporation and Red recombinase-mediated, homologous recombination. Step 3: The intermediate construct is isolated by selection on Km plates. Step 4: Overlapping extension PCR is used to construct PCR-B, with a foreign epitope sequence flanked by lamB regions as in PCR-A. Step 5: Co-electroporation of pBC-I-SceI and PCR-B. Here PCR-B is integrated into the genome by homologous recombination to replace the I-SceI recognition sequence and Km^r ^gene. Step 6: *S. enteritidis *containing the foreign epitope sequence is isolated by a counter-selection technique utilizing the pBC-I-SceI plasmid which expresses the I-SceI enzyme and Cm^r ^gene.

The goal of this study was to devise an efficient strategy to make markerless, site-directed mutations on a bacterial genome, using *S. enteritidis *as a model organism. The experimental method made use of overlapping extension PCR, the Red recombinase system, and an intermediary insertion of the I-SceI endonuclease recognition site as a counter-selection marker. The overall strategy is shown in Figure 1. Overlapping extension PCR was used to produce linear DNA with long flanking homology to the genome. The Red recombinase system was used to mediate recombination between incoming linear, PCR-generated DNA with the bacterial genome. In the two-step mutation process, the I-SceI site/kanamycin resistance (Km^r^) cassette was first inserted into the chromosome in the *lamB *gene by homologous recombination. Then, this mutation was replaced with the desired insertion sequence (LM2, CD154s, combination sequence I or combination sequence II). To make the replacement, a PCR product carrying the desired insertion sequence was added simultaneously with a plasmid encoding the I-SceI endonuclease enzyme used for counter-selection between the first and second mutations.

### I-SceI site/Km^r ^insertion mutation

The first mutation step involved designing a PCR fragment, PCR-A, which would serve as the carrier of the I-SceI site/Km^r ^cassette to be inserted into the *lamB *site. PCR-A consisted of the I-SceI enzyme recognition site adjacent to the Km^r ^gene with approximately 200–300 bp of flanking DNA on each end homologous to the upstream and downstream regions of *lamB *loop 9 insertion site (loop 9 up and loop 9 down, respectively). The fragment was introduced into *S. enteritidis *cells expressing Red recombinase enzymes. This step proved to be straightforward, and selecting for Km^r ^colonies was the first criteria for identifying potential positive clones. After screening a few colonies by colony PCR, positive clones were sequenced for the desired inserted I-SceI site/Km^r ^sequence, and the identified mutants in *S. enteritidis *were designated SE164.

### Genomic replacement of I-SceI/Km^r ^with LM2, CD154s, combination sequence I or combination sequence II

**Figure 2:**
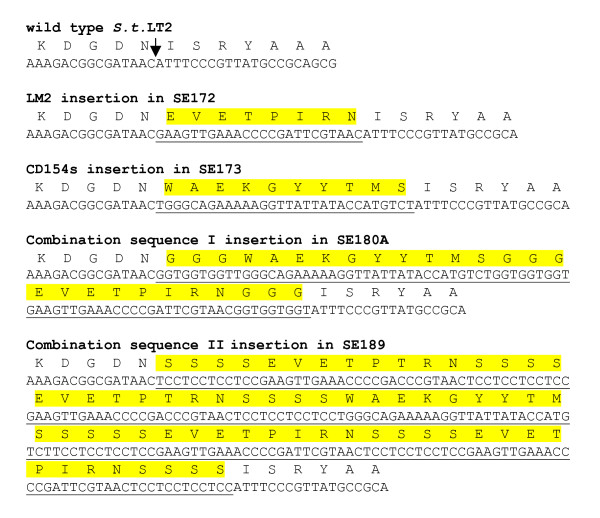
Translated sequences of insertion and surrounding lamB region; Shown here are translated regions corresponding to nucleotides 1243–1278 of the *lamB *gene of *Salmonella typhimurium *LT2 [16] and foreign insertion sequences placed in the homologous regions of *lamB *in *Salmonella enteritidis*. Underlined nucleotides represent inserted sequences as named, and the predicted corresponding amino acids are highlighted. The arrow shows the site in wild type which corresponds to the insertion site of mutants.

The second mutation step required constructing a PCR fragment, referred to as PCR-B, consisting of the final insertion sequence (LM2, CD154s, combination sequence I or combination sequence II) flanked by *lamB *homologous fragments. PCR-B amplicons have no selection marker and must be counter-selected after replacement for the previous I-SceI site/Km ^r ^mutation in SE164. Plasmid pBC-I-SceI encodes the chloramphenicol resistance (Cm^r^) gene and the I-SceI enzyme, which will cut the genome at the I-SceI site of SE164. Therefore, pBC-I-SceI was electroporated into SE164 along with PCR-B. After recombination of PCR-B to replace PCR-A, positive clones were chosen based on the ability to grow on chloramphenicol (Cm) but not on kanamycin (Km). After DNA sequencing of mutants to confirm successful recombination of PCR-B into SE164, the strains were designated SE172, SE173, SE180A, and SE189 for insert sequences LM2, CD154s, (Gly)_3_-CD154s-(Gly)_3_-LM2-(Gly)_3_, and (Ser)_4_-M2eA-(Ser)_4_-M2eA-(Ser)_4_-CD154-(Ser)_4_-LM2-(Ser)_4_-LM2-(Ser)_4_, respectively. For electroporation of LM2 and CD154s PCR-B products, at least 600 colonies were Cm^r^. To test efficiency of counter-selection, fifty Cm^r ^colonies from each the LM2 and the CD154s electroporation reaction were screened by restreaking on Km plates. For both LM2 and CD154s clones, 96% of each proved to be Cm^r ^but Km sensitive (Km^s^). Ten random clones for each the LM2 and CD154s insertion were used for PCR with lam 3f and lam 3r then digested using unique restriction enzymes sites for each insertion sequence. 100% of clones tested by digestion were positive for the desired mutation sequence, concluding the reliability and high efficiency of this selection scheme. Sequencing results in Figure 2 prove the replacement of the new sequence (LM2, CD154s, combination sequence I or combination sequence II) exactly into the loop 9 region without the addition of extraneous nucleotides.

### LM2 antibody responses in chickens

**Figure 3:**
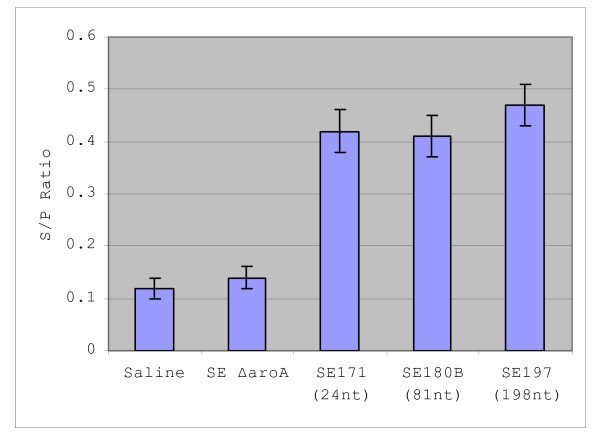
LM2 antibody responses in chickens; Results of chicken LM2 antibody responses to oral gavage challenge with saline, *S.enteritidis *Δ*aroA *(SE Δ*aroA*), SE171, SE180B and SE197 are shown. If the challenge strain carries an inserted epitope in the loop 9 region of *lamB*, the length in number of mucleotides (nt) is indicated.

After introducing attenuating mutations in the *aroA *gene of SE172 and SE180A and in the *aroA *and *htrA *genes of SE189, the resulting strains were designated SE171, SE180B and SE197, respectively. LM2 antibody responses were analyzed using serum from chickens challenged with saline, *S. enteritidis *Δ*aroA*, SE171, SE180B or SE197. ELISA results showed 3.00, 2.92 and 3.36 fold increases for SE171, SE180B and SE197, respectively in production of LM2 antibodies compared to *S. enteritidis *Δ*aroA *alone (Figure 3). This clearly indicates the ability of the inserted epitopes to be properly expressed.

## Conclusion

Here, we have reported scarless, site-directed mutagenesis performed in *S. enteritidis*. The described protocol proves the ability of making exact mutations by inserting short sequences ranging from 24–198 nucleotides, without any excess sequence, into the *lamB *gene of *S. enteritidis *genome. The basis for this method, which uses PCR products consisting of long homologous fragments flanking an insertion sequence along with the functions of Red recombinase, helps dodge the *S. enteritidis *restriction system. More significantly, we have provided a means to quickly produce bacteria expressing heterologous epitopes on the cell surface, which can then be tested for vaccine potential. This scarless, site directed mutation strategy overcomes the difficulties of chromosomal mutation and is simple in the design and methods used. First, primers for overlapping PCR must be designed. Then conventional techniques using PCR, DNA purification and electroporation can complete the project. Electroporation results as well as screening for mutants on antibiotic media are effective and convenient. One potential drawback of the experimental design is that the final mutants will still carry the pBC-I-SceI plasmid which has the Cm^r ^gene. To make this mutational strategy more significant for vaccine development, constructing a temperature-sensitive plasmid expressing I-SceI enzyme to replace pBC-I-SceI would allow for a final bacterial vector free of antibiotic genes. However, we found that pBC-I-SceI is not stable in the cells upon infection of chickens. When we recover these strains after vaccination of chickens, usually most of them do not retain Cm resistance, indicating frequent loss of the plasmid during infection. Such colonies isolated and verified to have lost pBC-I-SceI could be considered directly as vaccine candidates. On the other hand, an alternative method has been described which uses suicide plasmids to introduce simultaneously the foreign insert sequence plus the I-SceI site and I-SceI enzyme [5]. The disadvantage is that these plasmids result in wild type and mutant alleles causing decreased efficiency of positive mutant selection. Also, extra cloning is required for plasmid construction, and a modular intermediate, allowing for various epitope insertions, would not be available due to the design scheme.

When creating insertion mutations, the length, nature and location of the inserted sequence will be a factor of proper expression and sustained fitness of the organism. A possible hindrance in expressing foreign DNA in bacterial cells is the unknown ability of the organism to properly express functional eukaryotic proteins or peptides in the same fashion as the native organism. When applicable, our strategy of inserting into the genome short, foreign epitope sequences, which have been codon-optimized, as a fusion protein may largely circumvent this potential problem. Currently, we cannot confirm an upper limit on the added nucleotide capacity of loop 9 but are continuing to make more mutations with longer sequences. We have already successfully inserted sequences of length 24, 30, 81 and 198 nucleotides in this loop 9 region, yet experimental results seem to indicate that longer inserts may have decreased the invasive ability of *S. enteritidis *during chicken infection (data not shown). On the other hand, chicken serum antibody responses testing the *in vivo *expression of all ranges of insertion sequences have shown significantly increased LM2-specific antibody titers in chickens challenged with the epitope mutants compared to those challenged with saline or *S. enteritidis *Δ*aroA *alone (Figure 3). Experiments are underway to further characterize the immunogenic potential of the vaccine strains developed in this study.

Finally, this site-directed, scarless genomic mutagenesis strategy has opened up many opportunities to manipulate and examine the genome of *S. enteritidis *with acute specificity. The possibilities for vaccine prospects include targeting other genes of interest with immunogenic flagging potential as has been done with flagellin and secreted proteins [11,12]. Also, live attenuated vaccines can be produced by conducting scarless deletions of virulence genes.

Previously, it was demonstrated that transposons, containing the Km^r ^gene and the Sce-I site, could be inserted into random chromosomal locations, including 1976 genes [6]. Therefore, the Sce-I site could serve well as a counterselection marker, replacing altered alleles regardless of the location, directly supporting the feasibility of this newly described mutagenesis protocol working in other chromosomal contexts as well. Thus, this protocol could be used to explore other areas of research by adding epitope tags to proteins, adding or deleting promoters to assess gene contribution and function or creating site-specific insertion or deletion mutations. Furthermore, this method for conducting scarless, site-directed mutations has potential in other gram-negative bacteria and would be especially helpful for those organisms, other than *E. coli*, which may also need long sequence homology for efficient recombination.

## Methods

### Strains and Culture Conditions

All plasmids were first maintained in TOP10 *E. coli *cells (Invitrogen, Carlsbad, CA, USA) unless described otherwise. *Salmonella enteritidis *13A [13] was used for introduction of mutations. Bacteria carrying plasmid pKD46 were grown at 30°C, but growth of all other bacteria as well as plasmid curing was done at 37°C.

Luria-Bertani (LB) mediawas used for routine growth of cells, and SOC media (Invitrogen, Carlsbad, CA, USA) was used for phenotypic expressi on after electroporation. When appropriate, the following antibiotics were added to the media: ampicillin (Amp) at 100 μg/ml, kanamycin (Km) at 50 μg/ml, and chloramphenicol (Cm) at 25 μg/ml.

### Plasmids

Plasmids pKD46, pKD13, and pBC-I-SceI used for the present study were described previously [2,6]. Plasmid pKD46 encodes Red recombinase enzymes which mediate homologous recombination of incoming linear DNA with chromosomal DNA. This plasmid also contains the Amp resistance gene and is temperature-sensitive so that it requires 30°C for maintenance in the cell. Plasmid pKD13 served as a template for amplification of the Km resistance (Km^r^) gene used in overlapping PCR. Plasmid pBC-I-SceI produces the I-SceI enzyme, which cleaves the following 18 base pair, rare recognition sequence: 5'-TAGGGATAACAGGGTAAT-3' [14]. Also, the chloramphenicol resistance (Cm^r^) gene is located on pBC-I-SceI, and this plasmid can be maintained in the cell at 37°C.

### PCR

All primers used for PCR are listed in Table 1. Typical PCR conditions consisted of approximately 0.1 μg of purified genomic, plasmid or PCR-generated DNA (Qiagen, Valencia, CA, USA), 1× cloned *Pfu *polymerase buffer, 5 U *Pfu *polymerase (Stratagene La Jolla, CA, USA), 1 mM dNTPs (GE Healthcare Bio-Sciences Corp., Piscataway, NJ), 1.2 μM each primer in a total volume of 50 μL. The DNA engine thermal cycler (Bio-Rad, Hercules, CA, USA) was used with the following amplification conditions: 94°C for 2 minutes; 30 cycles of 94°C sec for 30 sec, 58°C for 60 sec, 72°C for 90 sec per 1 kb; and 72°C for 10 minutes for final extension. Each PCR product was gel purified (Qiagen, Valencia, CA, USA) and either eluted in 25 μL EB buffer for preparation of templates used in overlapping extension PCR or in 50 μL EB buffer, ethanol precipitated and suspended in 5 μL of ddH_2_O for electroporation into *S. enteritidis*.

**Table 1:** Primer sequences

**Primer**	**Amplified region**	**Primer sequence**
lam-up-f	loop 9 up	5'TGTACAAGTGGACGCCAATC 3'
lam-up-r		5'*GTTATCGCCGTCTTTGATATAGCC *3'

lam-dn-f	loop 9 dn	5'*ATTTCCCGTTATGCCGCAGC *3'
lam-dn-r		5'GTTAAACAGAGGGCGACGAG 3'

Km-f	I-SceI/Km^r ^gene	5'*GCTATATCAAAGACGGCGATAAC ***TAACTATAACGGTCCTAAGGTAGCGAAT**TTCCGGGGATCCGTCGA 3'
Km-r		5'*GCTGCGGCATAACGGGAAAT *TGTAGGCTGGAGCTGCTTCG 3'

Kan4f	inside Km^r ^gene: sequencing	5'CAAAAGCGCTCTGAAGTTCC 3'
Kan4r		5'GCGTGAGGGGATCTTGAAGT 3'

lam-i1	M2e/loop 9 dn	5'*GCTATATCAAAGACGGCGATAAC *GAAGTTGAAACCCCGATTCGTAAC*ATTTCC CGTTATGCCGCAGCG *3'

lam-i2	CD154s/loop 9 dn	5'*GCTATATCAAAGACGGCGATAAC *TGGGCAGAAAAAGGTTATTATACCATGTCT*ATTTCCCGTTATGCCGCAGC *3'

i2-i1h-f	CD154s-(Gly)_3_-LM2-(Gly)_3_-loop 9 dn	5'TGGGCAGAAAAAGGTTATTATACCATGTCTGGTGGTGGTGAAGTTGAAACCCCGATTCGTAACGGTGGTGGT*ATTTCCCGTTATGCCGCAGC *3'

i2-i1-r	CD154s-(Gly)_3_-loop 9 Up	5'AGACATGGTATAATAACCTTTTTCTGCCCAACCACCACC*GTTATCGCCGTCTT TGATATAGCC *3'

TJ1-f	CD154-(Ser)_4_-LM2-(Ser)_4_-LM2-(Ser)_4_-loop 9 dn	5'TGGGCAGAAAAAGGTTATTATACCATGTCTTCCTCCTCCTCCGAAGTTGAAACCCCGATTCGTAACTCCTCCTCCTCCGAAGTTGAAACCCCGATTCGTAACTCCTCCTCCTCC*ATTTCCCGTTATGCCGCAGC *3'

TJ1-r	CD154-(Ser)_4_-M2eA-(Ser)_4_-M2eA-(Ser)_4_-loop 9 up	5'AGACATGGTATAATAACCTTTTTCTGCCCAGGAGGAGGAGGAGTTACGGGTCGGGGTTTCAACTTCGGAGGAGGAGGAGTTACGGGTCGGGGTTTCAACTTCGGAGGAGGAGGA*GTTATCGCCGTCTTTGATATAGCC *3'
lam 3f	outer regions of loop 9: sequencing	5'GCCATCTCGCTTGGTGATAA 3'
lam 3r		5'CGCTGGTATTTTGCGGTACA 3'

aroA-1F	*aroA *up	5'CTGGACGTCTCTCGCTATGG 3'
aroA-1R		5'TAGGAACTTCGAAGCAGCTCCAGCCTACACATAAAAACC CCACAGACTGG 3'

aroA-2F	*aroA *dn	5'GGAATAGGAACTAAGGAGGATATTCATATGGTCTTCTGTTGCGCCAGT 3'
aroA-2R		5'CTTGCGAGAGTGCCCTAAAG 3'

htrA-1F	*htrA *up	5'GGTTTTAGCCGCCTGCTT 3'
htrA-1R		5'TAGGAACTTCGAAGCAGCTCCAGCCTACACTTGCTGTGTACGTCAGATTCA 3'

htrA-2F	*htrA *dn	5'GGAATAGGAACTAAGGAGGATATTCATATGTCACCTTTGTCCCCCTTC 3'
htrA-2R		5'GCATCATTTCGGCAGTCATA 3'

Kan 3F	Km^r ^gene	5'GTGTAGGCTGGAGCTGCTTC 3'
Kan 3R		5'CATATGAATATCCTCCTTAG 3'

Italicized nucleotides are those which have complementation to either side of the *lamB *gene loop 9 insertion site, which corresponds to nucleotide 1257 using *S. typhimurium *as an annotated reference genome. Bold font nucleotides represent the I-SceI site in the Km-f primer, and underlined sequences are all other insert sequences.

### Electroporation

Transformation of pKD46 into *S. enteritidis *was the first step carried out so that Red recombinase enzymes could be used for mediating recombination of subsequent mutations.

Plasmid pKD46 was harvested from *E. coli *BW25113 [2] using a plasmid prep kit (Qiagen Valencia, CA, USA). Then 0.5 μL of pKD46 DNA was used for transformation into *S. enteritidis *13A which had been prepared for electroporation according to a previously described protocol with few modifications [2]. Briefly, cells were inoculated into 10–15 mL of 2X YT broth and grown at 37°C overnight. Then 100 μL of overnight culture was re-inoculated into 10 mL fresh

2X YT broth at 37°C for 3–4 hours. Cells to be transformed with pKD46 plasmid were heated at 50°C for 25 minutes to help inactivate host restriction [15]. Cells were washed five times in ddH_2_O water and resuspended in 60 μL of 10% glycerol. Cells were then pulsed at 2400–2450 kV for 1–6 ms, incubated in SOC for 2–3 hours at 30°C and plated on LB media with appropriate antibiotics. *S. enteritidis *transformants with pKD46 were maintained at 30°C. When these transformants were prepared for additional electroporation reactions, all steps were the same except that 15% arabinose was added to induce Red recombinase enzymes one hour prior to washing, and cells did not undergo the 50°C heat step.

### Loop 9 up- I-SceI/Km^r^- Loop 9 down Construct

**Figure 4:**
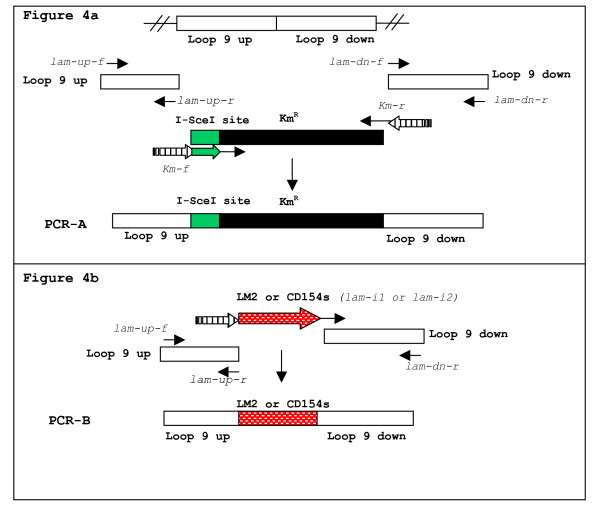
**4a and 4b**; Design scheme of PCR-A and PCR-B products used for introducing mutations; Figure 4a shows the design and process of amplification of PCR-A product which consists of the I-SceI recognition site (I-SceI site), Km^r ^gene, and 200–300 bp of flanking homology to the loop 9 region of the lamB gene (loop 9 up and loop 9 down). Figure 4b is representative of the PCR-B product of LM2 and CD154s only, which consists of either the LM2 or CD514s sequence flanked by the same loop 9 regions as in PCR-A. Striped arrows indicate the section of the primer sequences that are complementary to loop 9 regions and thus are part of the homology overlap in PCR reactions. I-SceI site and epitope sequences which have been added to primer sequences are represented by arrows with the same pattern as the corresponding section in this DNA diagram. All primer sequences are italicized.

Introduction of I-SceI enzyme recognition site along with the Km^r ^gene into loop 9 of the *lamB *gene was done using the Red recombinase system and overlapping PCR as described previously [2,8]. The insertion site corresponds to nucleotide 1257 of the *lamB *gene using *Salmonella typhimurium *LT2 (S.*typhimurium*) as an annotated reference genome [16]. First, the upstream and downstream regions immediately flanking the loop 9 insertion site (loop 9 up and loop 9 down, respectively) were amplified separately. Primers used were lam-up-f and lam-up-r for loop 9 up and lam-dn-f and lam-dn-r for loop 9 down. Then the Km^r ^gene from pKD13 plasmid [2] was amplified using primers Km-f and Km-r. Here, the I-SceI enzyme site was synthetically added to the 5' end of Km-f primer then preceded by a region complimentary to the loop-up-r primer. Likewise, a region complimentary to the loop-dn-f primer was added to the 5' end of Km-r primer. The complimentary regions allow all 3 PCR products to anneal when used as templates in one PCR reaction. Figure 4a represents this design scheme. PCR fragments consisting of loop 9 up- I-SceI/Km^r^- loop 9 down sequence (PCR-A) were electroporated into *S. enteritidis*, which harbored pKD46 and were induced by arabinose, and then plated on LB with Km plates. To verify the correct sequence orientation of the mutation, we performed colony PCR with primer pairs Kan4F/lam3f and Kan4R/lam3r, where Kan4F and Kan4R are Km^r ^gene-specific primers and lam3f and lam3r are primers located outside the *lamB *loop 9 region. These PCR fragments were gel purified (Qiagen, Valencia, CA, USA) and used for DNA sequencing. The verified mutant which carried the I-SceI/Km^r ^fragment in the mentioned loop 9 region of the *lamB *gene was designated SE164.

### Loop 9 up- LM2, CD154s, combination sequence I or combination sequence II- loop 9 down construct

The final overlapping PCR fragment, PCR-B, contained the added LM2, CD154s, combination sequence I or combination sequence II flanked by loop 9 up and down regions (Figure 4b). Combination sequences consisted of LM2 or an alternate M2e epitope associated with avian species (M2eA) [17] and CD154s along with spacers such as Glycine (Gly) or Serine (Ser) residues. Inserted sequences were as follows: LM2: GAAGTTGAAACCCCGATTCGTAAC; CD154s:

TGGGCAGAAAAAGGTTATTATACCATGTCT; combination sequence I (Gly)_3_-CD154s-(Gly)_3_-LM2-(Gly)_3_:

GGTGGTGGTTGGGCAGAAAAAGGTTATTATACCATGTCTGGTGGTGGTGAAGTTGAAACCCCGATTCGTAACGGTGGTGGT; and combination sequence II (Ser)_4_-M2eA-(Ser)_4_-M2eA-(Ser)_4_-CD154-(Ser)_4_-LM2-(Ser)_4_-LM2-(Ser)_4_:

TCCTCCTCCTCCGAAGTTGAAACCCCGACCCGTAACTCCTCCTCCTCCGAAGTTGAA ACCCCGACCCGTAACTCCTCCTCCTCCTGGGCAGAAAAAGGTTATTATACCATGTCT TCCTCCTCCTCCGAAGTTGAAACCCCGATTCGTAACTCCTCCTCCTCCGAAGTTGAA ACCCCGATTCGTAACTCCTCCTCCTCC.

To shorten the amount of steps for construction of this next fragment, the LM2 or CD154s sequence was synthetically added to the 5' end of the lam-dn-f primer and preceded by the complimentary region to the loop-up-r primer. The previously used PCR product for loop 9 up could be used together with the newly constructed PCR product in which LM2 or CD154s were incorporated at the 5' end of loop 9 down to perform the final PCR reaction. However, for other insert sequences (referred to as combination sequence I and II), an extra PCR step was needed, due to the longer lengths of insert sequences, to amplify loop 9 up with added nucleotides specific to insertion sequences connected to loop-up-r primer. The coding sequence for Gly (GGT) and Serine (TCC) as well as all other amino acids were chosen based on compiled data of the most frequently used codons in *E. coli *and *Salmonella typhimurium *proteins [18]. See Table 1 for further details of primer design.

### Genomic replacement of I-SceI/Km^r ^with LM2, CD154s, combination sequence I or combination sequence II

PCR-B products and plasmid pBC-I-SceI (at a molar ratio of approximately 40:1) [6] were simultaneously electroporated into SE164 cells, which carried the I-SceI/Km^r ^fragment in loop 9 of the *lamB *gene. Clones for each PCR-B recombination mutation were chosen according to the ability to grow on Cm plates but not on Km plates, due to the replacement of PCR-B for the Km^r ^encoding PCR-A sequence. Modified regions in the selected clones were PCR-amplified, and DNA sequences were determined using primers lam3f and lam3r located outside the loop 9 down and up amplified regions. The assigned strain numbers for epitope insertions LM2, CD154, combination sequence I and combination sequence II were SE172, SE173, SE180A and SE189, respectively.

### Creating attenuating mutations in *aroA *and *htrA *genes

Bacteria carrying the mentioned epitope sequences were attenuated so that vaccine potential and epitope expression could be assessed. Attenuation was done by inserting the Km^r ^gene in place of and thereby deleting *aroA *and/or *htrA*. This was done by first using overlapping PCR with primers aroA-1F, aroA-1R, aroA-2F and aroA-2R for the *aroA *deletions and htrA-1F, htrA-1R, htrA-2F and htrA-2R for the *htrA *deletions. The Km^r ^gene was amplified from pKD4 using primers Kan 3F and Kan 3R (Table 1). Additionally, the previously described pCP20 was introduced into Δ*aroA *cells to remove the Km^r ^gene from the *aroA *gene only when the *htrA *gene was also to be deleted [2]. Mutations were made in the *aroA *gene of SE172, SE173, SE180A, and in the *aroA *and *htrA *genes of SE189 with the resulting strain numbers being SE171, SE174, SE180B and SE197, respectively.

### Challenge of *S. enteritidis *mutants in chickens

At day of hatch, broiler chicks were challenged via oral gavage with saline or 10^7 ^CFU of *S. enteritidis *Δ*aroA*, SE171, SE180B or SE197. Blood samples were taken at day 20 post-hatch to test for LM2 specific IgG antibody titers.

### Measuring LM2 antibody titers

LM2 antibody responses in challenged chickens were calculated using an antigen capture ELISA. Briefly, LM2 conjugated to BSA was added to a 96 well microtiter plate and allowed to incubate overnight at 4°C. Plates were then rinsed and incubated with chicken serum for 2 hours.

Then, plates were rinsed and a detection antibody was added and incubated for an additional 1 hour. Afterwards, plates were again rinsed, and a peroxidase substrate kit was used to obtain absorbance readings at 450 nm with a spectrophotometer. Positive and negative controls were included which consisted of the LM2 polyclonal antibody mentioned above and serum from an untreated bird, respectively. All samples were measured, and sample to positive control ratios (S/P ratios) were calculated using the following equation: (sample- negative control)/(positive control – negative control).

## Competing interests

The author(s) declares that there are no competing interests.

## Authors' contributions

MC is the main author of the manuscript as well as the individual who carried out the majority of experimental methods and part of the experimental design. SL, TJ and KC have also contributed to lab work as well as editing of the manuscript. WB, LB, BH and YMK are the Principal Investigators who gathered the concepts of experimental design. All authors have read and approved the final manuscript.
